# Surgical Orthodontic Treatment of an Impacted Canine in the Presence of Dens Invaginatus and Follicular Cyst

**DOI:** 10.1155/2014/643082

**Published:** 2014-05-21

**Authors:** Marialuce Spallarossa, Carola Canevello, Francesca Silvestrini Biavati, Nicola Laffi

**Affiliations:** ^1^Private Practice, Genoa, Italy; ^2^Orthodontic Post-Graduate Program, Dipartimento di Chirurgia e Scienze Stomatologiche, University of Cagliari, Via Binaghi 4/6, 09121 Cagliari, Italy; ^3^S.C. Odontostomatologia, E.O. Galliera Hospital, Via Mura delle Cappuccine 14, 16128 Genoa, Italy

## Abstract

*Introduction*. “Dens invaginatus” is a dental anomaly which originates from the invagination of the ameloblastic epithelium into the lingual surface of the dental crown during the odontogenesis. It can cause early pulpal necrosis, abscesses, retention or dislocation of contiguous elements, cysts, and internal resorptions. It normally affects the upper lateral incisors. In the following study the authors will discuss the etiology, the physiopathology, and the surgical-orthodontic management of a rare case of impacted canine associated with dens invaginatus and follicular cyst, with the aim of highlighting the importance of taking any therapeutic decision based on the data available in the literature. *Case Report*. The present study describes a combined surgical-orthodontic treatment of an impacted canine associated with a lateral incisor (2.2) suffering from type III dens invaginatus with radicular cyst, in a 15-year-old patient. *Discussion*. When treating a dens invaginatus there are different therapeutic solutions: they depend on the gravity of the anomaly and on the association with the retention of a permanent tooth. The aesthetic and functional restoration becomes extremely important when performing a surgical-orthodontic repositioning.

## 1. Introduction

“Dens invaginatus” is a dental anomaly, described for the first time in 1859 by Socrates [1]. Over the years, it has been associated with many synonyms: dens in dentis, invaginated odontome, tooth inclusion, dentoid in dente, dilated gestant odontoma, and dilated composite odontoma. Each of these terms reflects a specific etiopathogenetic hypothesis; today the more correct term “dens invaginatus” is used because such anomaly derives from the invagination of the ameloblastic epithelium on the lingual surface of the tooth crown during odontogenesis.

Although there is not a solid consensus on the etiology, a number of assumptions have been formulated and accepted as follows:

- the growth pressure of the dental arch causes the buckling of the enamel organ [2],
- the dental lamina degenerates, with a rapid and aggressive proliferation of the enamel epithelium which invades the tooth germ [3],
- infection or trauma during ontogeny [4, 5],
- genetic factors: the lack of signalling proteins may be responsible for dental abnormalities (e.g., absence of arm chromosome 7q32 is associated with dens invaginatus and hypodontia) [6, 7].

The dens invaginatus may present more severe clinical cases and there are many classifications proposed, and the most widely used is that of Oehlers [8] who described three types of invaginations, reported in Table 1.

The most affected tooth appears to be the upper lateral incisors where the response is frequently bilateral (43% of all cases) [9], and the literature reports a lower number of invaginations of the central incisors [10], canines, and premolars [11].

This anomaly may cause early pulp necrosis (a few years after tooth eruption) [13, 14], abscess formation, retention or displacement of adjacent teeth, cysts [15–18], and internal resorption [19].

The few case reports in the literature describing the association between cystic lesion and dens invaginatus report neoformation of disembriogenetic nature or cysts of inflammatory nature; to the best of the authors' knowledge an association between follicular cysts and dens invaginatus has not yet been discussed. The follicular or dentigerous cyst develops from proliferation of the enamel organ remnant or reduced enamel epithelium. As with other cysts, expansion of the follicular cyst is related to an increase in cyst fluid osmolality and the release of bone-resorbing factors. These cysts are mainly related to the third upper molar, lower third molar, and maxillary canine which are the most commonly impacted teeth [20].

Cysts and dens invaginatus can be considered as local pathogenetic factors of dental inclusion (Table 2).

The purpose of this study is to present the therapeutic procedure of a rare case of microdontic dens invaginatus grade III associated with odontogenic cyst with inclusion of the ipsilateral canine.

## 2. Case Report

A 15-year-old boy was sent to the treating orthodontist at the S.C. of Dentistry E.O. “Ospedali Galliera” for the presence of dental anomaly of 2.2, inclusion of 2.3, and the presence of osteolitic lesion visible on RX OPT. His medical history was noncontributory.

**Figure 1:**
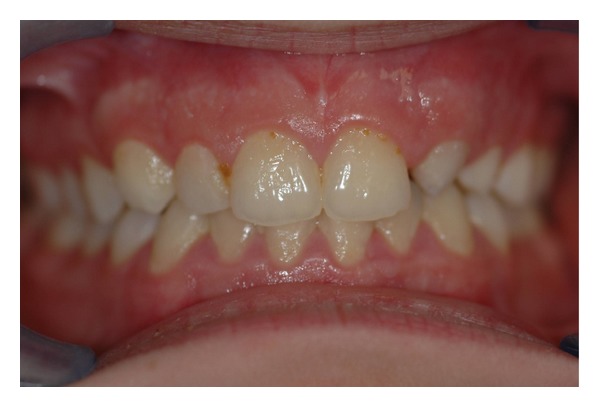


**Figure 2:**
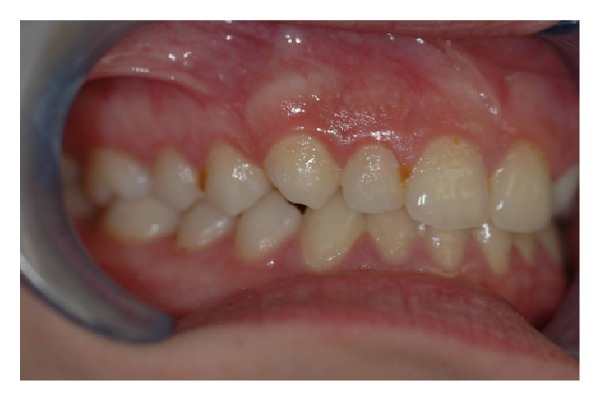


**Figure 3:**
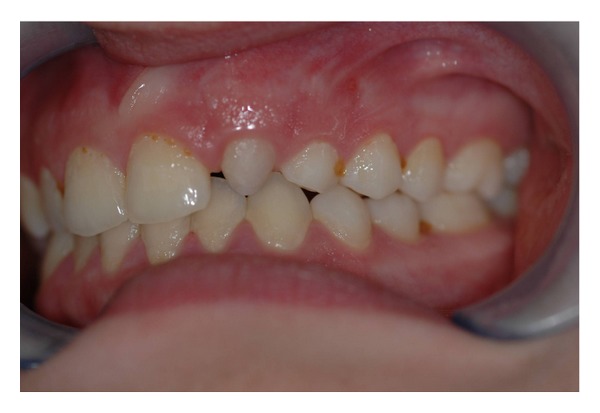


The intraoral examination revealed the anomaly of 2.2 microdontic, discolored, with absence of caries, and physiological periodontal probing. It also showed the absence of 2.3 and the presence of the deciduous 6.3. At the time of the visit, there were no symptoms and intraoral mucosa appeared to have no pathological signs. The thermal tests of vitality were positive for all dental elements with the exception of 2.2 (see Figures 1, 2, and 3).

**Figure 4:**
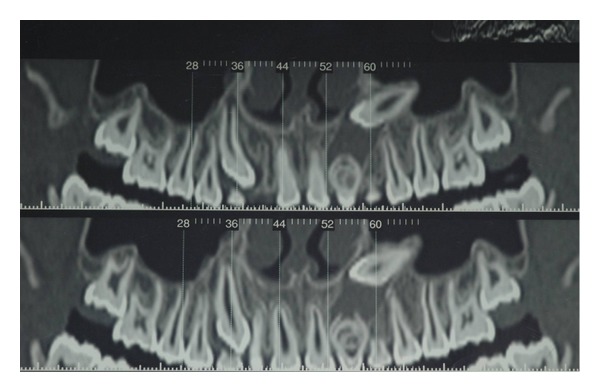


**Figure 5:**
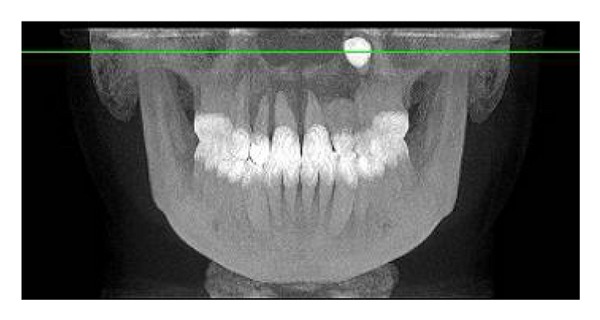


**Figure 6:**
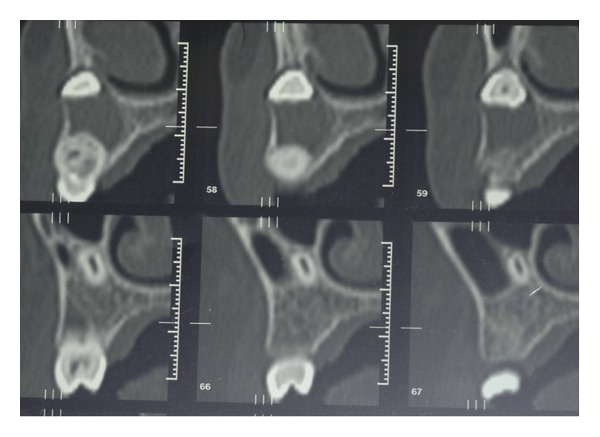


**Figure 7:**
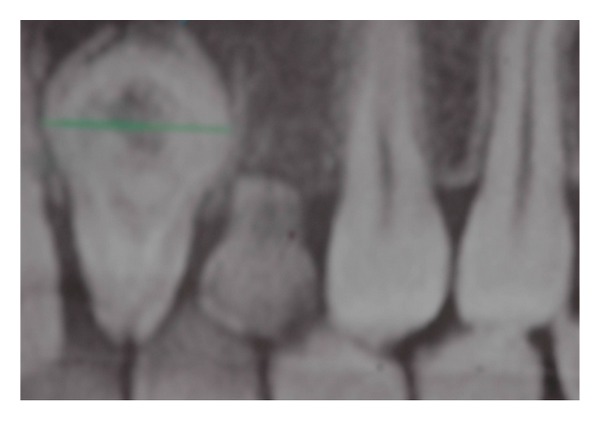


To complete the diagnosis a TC Dental Scan of the maxilla was carried out. This revealed dens invaginatus type III of 2.2 and unilocular radiolucency with corticated margins in association with the crown of 2.3 unerupted that was vestibulary displaced. This radiological features could correspond to odontogenic cyst but based on clinical appearance it was not possible to differentiate between radicular cysts and follicular cysts (see Figures 4, 5, 6, and 7).

Endodontic treatment of a necrotic dens invaginatus, given the serious anomaly, was ruled out; in case of major malformations, the extraction of the element itself is recommended [19, 21].

Treatment options for the impacted canine could be

1. surgical exposure of the tooth and its traction in the dental arch,
2. avulsion of the impacted tooth [22].

The angle of the root of the canine guaranteed a high predictability of orthodontic treatment. The risk of ankylosis of 2.3 had to be taken into consideration; however, the age of the patient was favorable. The patient and parents were informed of the possible treatment options. In the end, consensus was given for the combined surgical-orthodontic treatment and recovery of the impacted canine.

**Figure 8:**
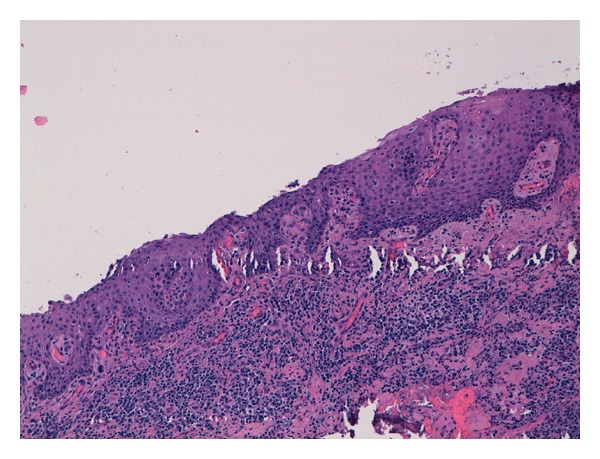


The surgical approach consisted of two distinct operations: one designed to enucleate the cyst and to bond the canine and the other dedicated to the extraction of the dens invaginatus and the deciduous canine. The same day of the cyst enucleation, a button and a metallic ligature was positioned on the canine to pull it out. A histological examination of the removed tissue was carried out: results showed the presence of stratified squamous and nonkeratinized epithelium. This is compatible with follicular odontogenic cyst (see Figure 8).

**Figure 9:**
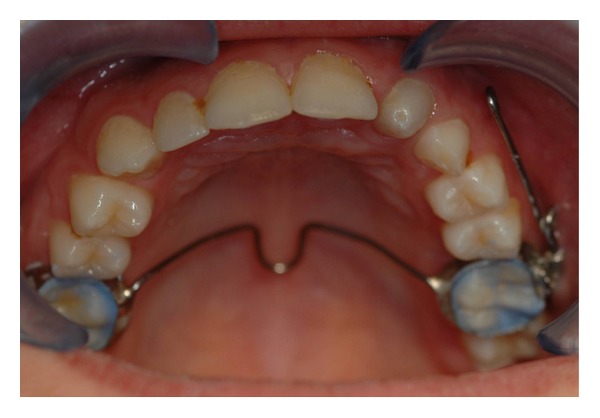


**Figure 10:**
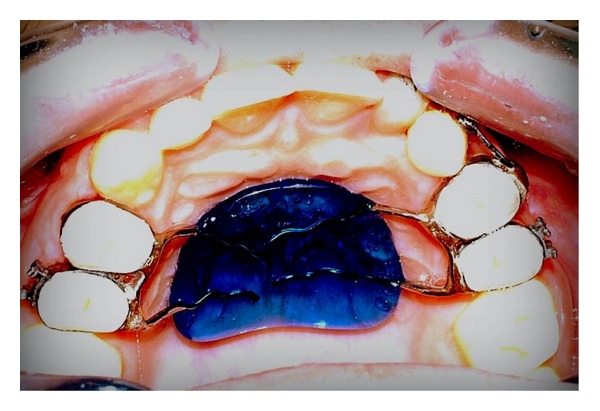


**Figure 11:**
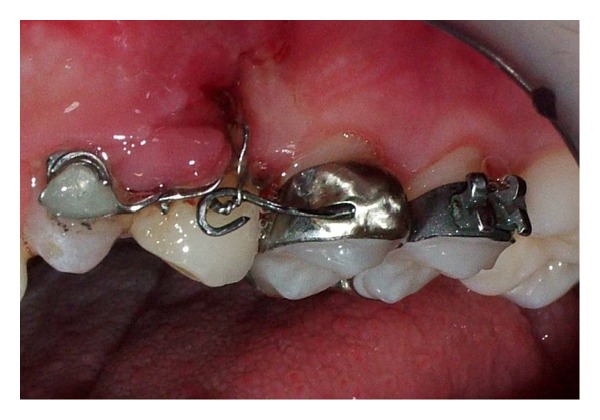


Considering the good alignement of the dental arches but, at the same time, being in need of a good anchorage system, it was decided not to completely bond the dental arches and to place a palatal arch with a Nance button. Tooth 2.2 was maintained in order to use it during the orthodontic traction (see Figures 9, 10, and 11).

**Figure 12:**
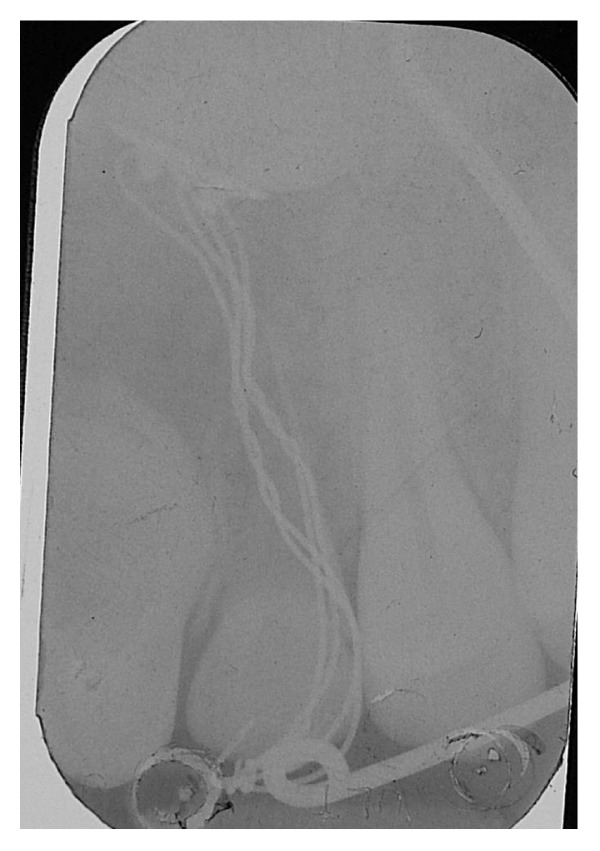


**Figure 13:**
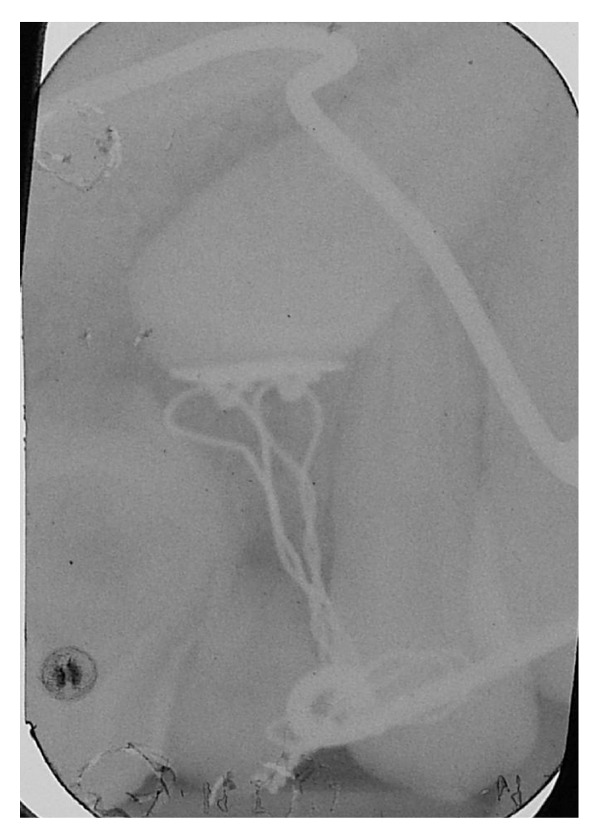


**Figure 14:**
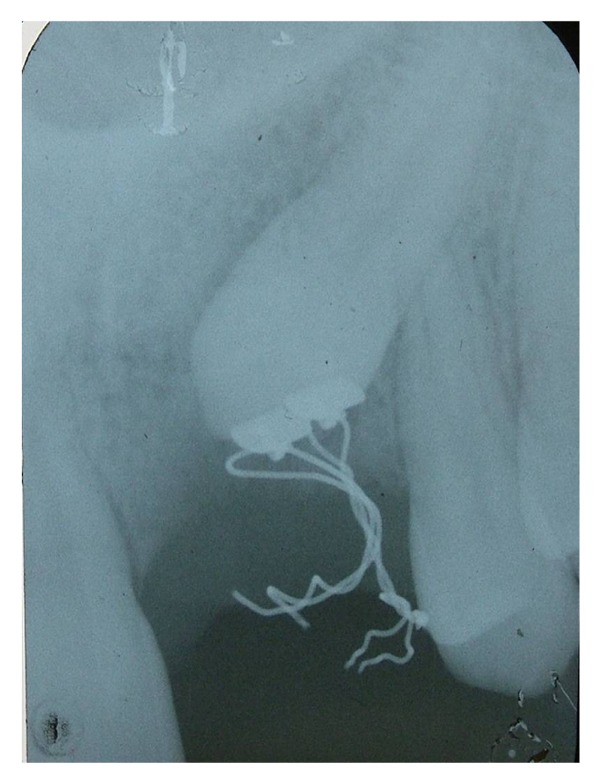


It can be considered as inconvenient to maintain a necrotic element, but given the reduced treatment times and the absence of symptoms associated with it, it was considered appropriate to accept the risk of inflammation rather than submit to the adiacent healthy teeth dangerous intrusive counterforce (root resorption) [23], which is inevitable during the traction of impacted canine. The metal ligature was activated every 20 days and periodically intraoral radiographs were made to check the eruption path of the canine (see Figures 12, 13, and 14).

**Figure 15:**
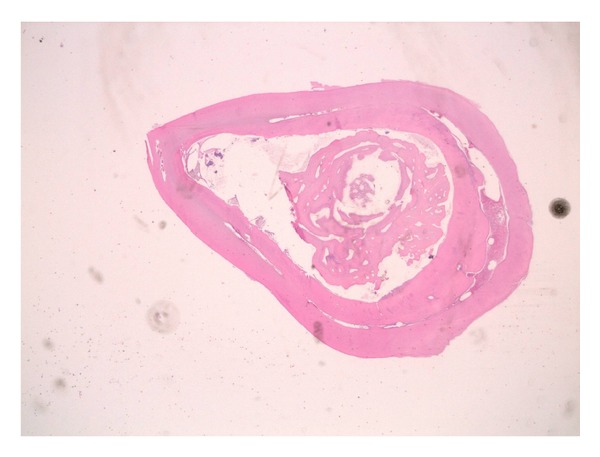


When the crown of the canine (2.3) had arrived in close proximity of the roots of teeth 2.2 and 6.3, avulsion of those teeth was performed (see Figure 15).

**Figure 16:**
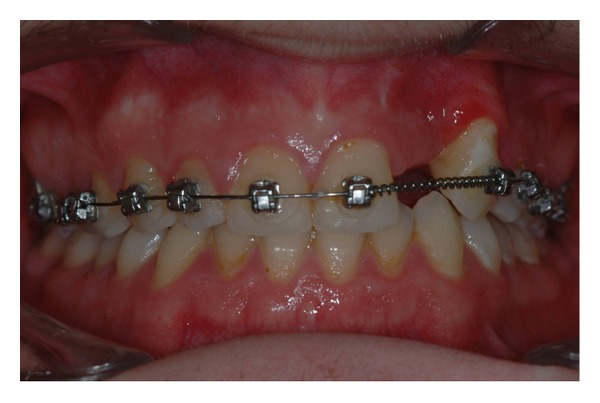


**Figure 17:**
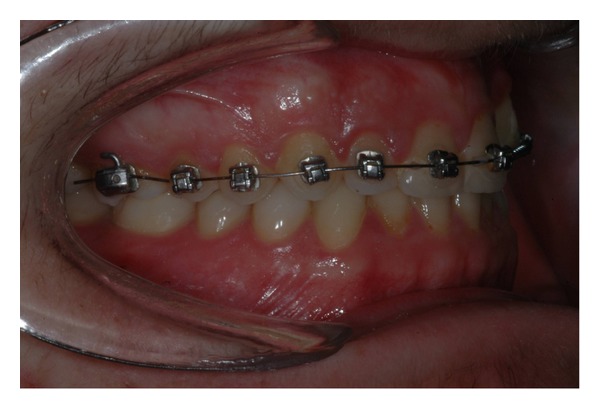


**Figure 18:**
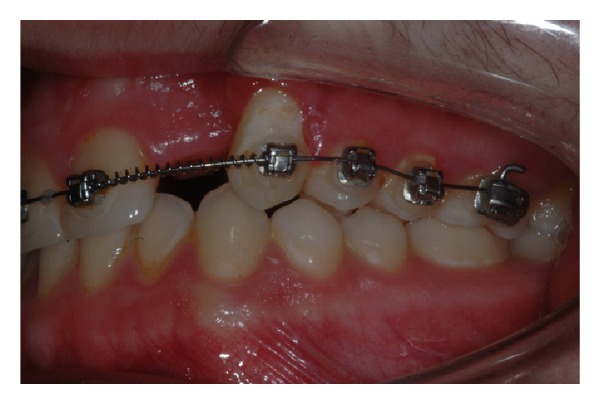


The following month, the arch was completely bonded: usual fixed multibrackets treatment steps were followed starting with alignment and levelling. Extended treatment times were not expected since the molar class I was maintained and the space in the dental arch was sufficient to accommodate the canine and the future implant prosthetic solution in area 2.2. The appliances used were low friction, straight wire bracket with Damon Q, which have led to a good occlusion (see Figures 16, 17, and 18).

**Figure 19:**
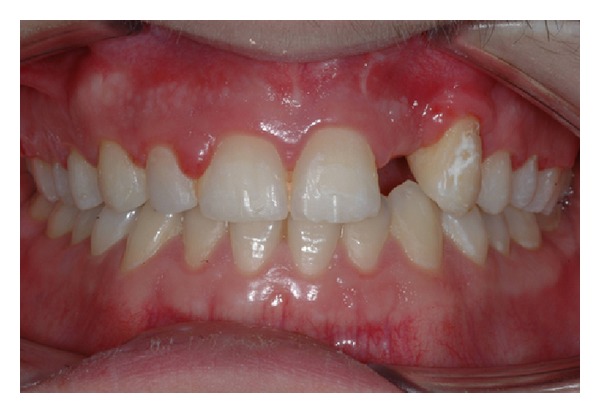


**Figure 20:**
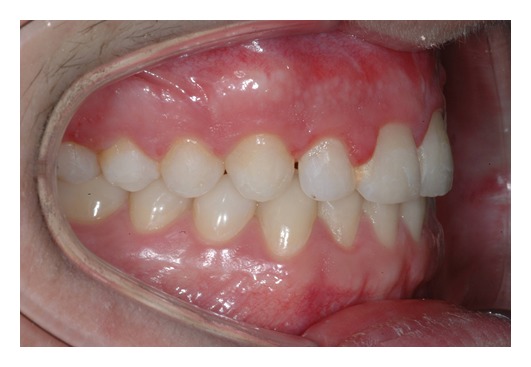


**Figure 21:**
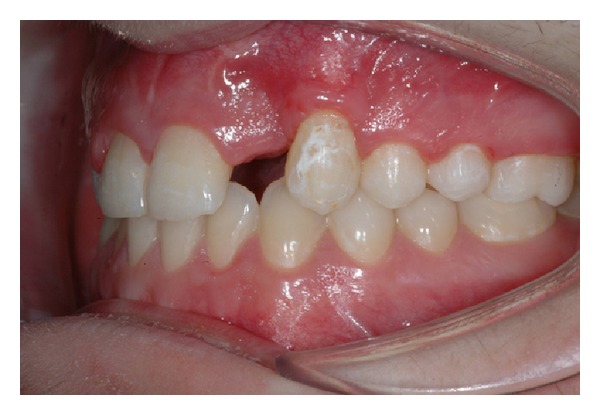


**Figure 22:**
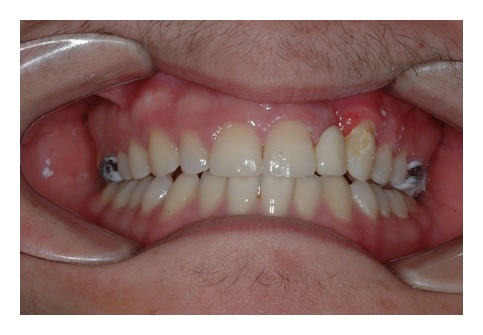


**Figure 23:**
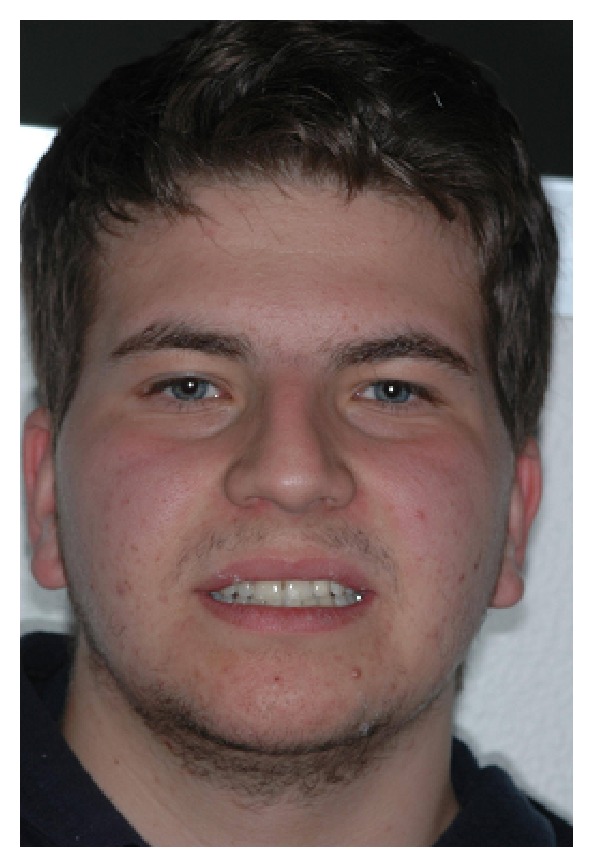


The patient was debonded after 18 months after the application of the brackets; a temporary Maryland bridge on the left lateral incisor was applied in order to give an aesthetic solution to the patient while waiting to proceed with the final implant-prosthetic restoration (the patient will wait until he is 22 years old) (see Figures 19, 20, 21, 22, and 23).

## 3. Discussion

In the literature (on permanent teeth) a prevalence of dens invaginatus that can vary from 0.3% to 10% is reported. Complications may be associated in 0.25%–26.1% of cases; the upper lateral incisor is the most affected element. This wide range of variability is due to the different study samples and diagnostic criteria used in several scientific works [24]. Deciduous teeth are rarely affected, as this represents a rare event in the microdontic teeth [25]. Invagination, whether there is a communication with the oral cavity, allows the passage of microorganisms and irritants that can reach the periodontium or the dental pulp through the thin layer of dentin and/or enamel that separates them [12]. This phenomenon often leads to necrosis of the pulp [13] and to consequent infectivous inflammatory events such as apical abscesses [14], parodontal abscesses [25], internal resorptions [19], or cysts [15–18].

In the event that the invagination does not communicate with the root canal system, the pulp can maintain his vitality [18, 26, 27]. Because of their altered anatomical structure, the endodontic treatment has little chance of success. For elements with sufficiently preserved coronal anatomy (type I Oehlser), the early radiological diagnosis and a prophylactic conservative treatment are essential to prevent pulp diseases. For more severe forms of dens invaginatus with overt infection of the pulp (types II and III of Oehlser) extraction is the normal treatment route [28].

Follicular cysts arise from the follicular sac of nonerupted teeth; at present little is known about the causes which lead to the separation of the epithelium from the enamel surface creating space for the accumulation of fluid around the crown of the included tooth. The growth of follicular cysts may be associated with bone destruction, displacement of adjacent teeth, and oronasal fistula [20, 29]. In this case, it is possible that germs and irritants have caused necrosis of the tooth and that they have achieved through the apex the follicular sac of the erupting 2.3 causing an inflammatory reaction that led to the origin of a cystic deviation.

Regarding the canine, different therapeutic options were available as follows: (1) waiting until the spontaneous eruption of the element after the extraction of the dens in dentis, (2) extraction of both canine and dens invaginatus with subsequent replacement of such elements with an implant-prosthetic solution, (3) reimplantation, and (4) surgical-orthodontic treatment. This solution was chosen since reimplanting is not very predictable (42% of reimplanted teeth have external root resorption within the first year) [30]. Also the spontaneous eruption of a fully formed element that does not have any thrust eruption seems very uncertain. An implant replacement in a very young patient must take into consideration the duration of such implant that could result later in biological and economic expense. In this case, having the canine as a good inclination [31] and therefore good predictability of success, it opted for traction in the arch with an orthodontic appliance.

During the initial therapy phase, a palatal Nance button was positioned to start the traction of the canine, keeping the abnormal tooth in the arch, employing it in traction. This was done not to involve the other teeth and to reduce the risk of root resorption resulting from intrusive counterforce that are inevitable during such movements [23]. Retaining tooth 2.2 also had a considerable aesthetic importance.

The second therapeutic phase began as soon as the crown of the canine came near to the roots of the deciduous tooth, planning the extraction of 2.2 and 6.3 and the complete bonding of the arch. It was decided to use a low friction systematic, with passive self-ligating brackets, Damon Q. This was chosen since the literature shows that this method is much more rapid during the phases of alignment and levelling, if compared to traditional methods [32]. It appears instead to be equivalent or even slower than other existing orthodontic techniques during the later stages of treatment, such as the working phase (gap closure, correct transverse-sagittal, and vertical relationships) and finishing phase. Our patient presented no problem in transverse, vertical, or sagittal relationships: it was in fact a dental and skeletal class I, without scissor or cross bites, standard overjet, and overbite. The use of a systematic which could be more effective in the most critical phases could have been more helpful.

It should be clarified that the type of prescription used was Damon Q brackets, available in three versions, low torque, standard torque, and super torque. The right prescription should be chosen according to the type of malocclusion, the possibility of using intra- or extraoral aids (elastics, headgear, transpalatal bar, etc.), or the inclination that you want to give to a dental element in particular. Since it was not designed to use elastics or other devices, it was decided to employ the standard torque on all teeth except the disincluded canine, for which we used a bracket with super torque. This decision was dictated by the fact that the canine still had a very buccally inclined root and to control that inclination we took advantage of the opportunity to use a bracket with a more positive torque.

The arches used were 0 : 14 thermal NiTi, 0 : 18 thermal NiTi, 0.14 × 0.25 thermal NiTi, 0.18 × 0.25 thermal NiTi, and 0.19 × 0.25 stainless steel. During the finishing stage, it was necessary to apply additional positive torque of 20° on the wire at the element 2.3 to incline the root towards the palate.

At the end of orthodontic treatment, a temporary Maryland bridge was applied on the missing element in order to wait until the patient reached 22 years of age to proceed with the implant-prosthetic restoration. Before that age, the literature argues negatively on the insertion of osseointegrated implants.

## 4. Conclusion

Treatment options for dens invaginatus are diverse and differ depending on the degree of abnormality; when this alteration occurs in association with retention of a permanent dental element, treatment strategies must be directed to the aesthetic and functional restoration by orthodontic-surgical recovery of the included tooth, with a multidisciplinary approach.

More importantly, before undertaking any therapeutic procedure, every possible treatment hypotheses and intervention strategies should be evaluated in light of what the literature suggests. And most of all, the clinician needs to study the predictability of success/failure for each technique used and to relate it to the clinical condition of the patient, with everything always supported by data accepted in the scientific field nationally and internationally.

All of this is designed to offer the patient the best solution to its occlusal disharmony in terms of comfort, aesthetics, function, and general well-being.

## Figures and Tables

**Table 1:** 

I.	The invagination of the ameloblastic line is minimal, confined within the crown of the tooth, and does not exceed the cementoenamel junction.

II.	Characterised by the invagination extending apically beyond the cementoenamel junction, where a connection between the invagination and the pulp is possible.

III.a	The invagination extends through the root and communicates laterally with the periodontal ligament space through a pseudoforamen. There is usually no communication with the pulp, which lies compressed within the root.

III.b	The invagination extends through the root and communicates with the periodontal ligament at the apical foramen. There is usually no communication with the pulp [12].

**Table 2:** 

General factors	Local factors
Prenatal (I) Hereditary predisposition (II) Chromosomal abnormalities (S. Down) (III) Embryopathies (LPS) (IV) Fetopathies (cleidocranial dysostosis) (V) Infectious diseases (Syphilis, rubella, scarlet fever, and toxoplasmosis) Postnatal (I) Disendocrine (hypothyroidism and hypopituitarism) (II) Diseases of malnutrition (hypovitaminosis) (III) Anemia	(I) Dentoalveolar/skeletal discrepancy (II) Anomalies of dental development in the load of the lateral incisors (agenesis, malposition, underdevelopment, etc.). (III) Abnormal position of the gem of the canine, ectopy, and transpositions (IV) Trauma in deciduous teeth (V) Prolonged retention or early loss of deciduous (VI) Ankylosis or premature closure of the apex of the canine (VII) Iatrogenic factors (VIII) Alveolar cleft (IX) Tumor formation, odontomas, and cysts supernumerary
